# Mitral supravalvular ring: a case report

**DOI:** 10.1186/1476-7120-3-19

**Published:** 2005-08-11

**Authors:** Walter Serra, Paola Testa, Diego Ardissino

**Affiliations:** 1 Heart Department, Cardiology Division, Azienda Ospedaliera/Universitaria Parma, Italy

**Keywords:** transesophageal echocardiography, mitral supravalvular ring

## Abstract

Supravalvular mitral stenosis is a rare condition characterized by an abnormal ridge, with one or two orifices, covering and obstructing the mitral valve. Preoperative diagnosis is difficult with transtoracic echo (TTE), angiography and magnetic resonance imaging (MRI).

In this case, a 36-year-old male, was admitted to our Heart department: He experienced progressive dyspnea on effort and at rest.

Diagnosis was made by transesophageal echocardiography which showed, on apical 4-chamber section, an anulare structure attached since a membrane to the atrial wall anterior mitral valve leaflet and just proximal to the posterior mitral leaflet.

Pre-operative identification of the supravalvular mitral ring is the target for obtaining good surgical results. Cineangiography and MRI both failed in reaching this objective, whereas, transesophageal echocardiography is the best method to identify this congenital heart disease.

Using TEE the identification is not only possible but also easier.

## Background

Mitral supravalvular ring is a rare congenital heart defect, as descibed by Fisher [1], characterized by an abnormal ridge of connective tissue on the atrial side of the mitral valve. Often the supravalvular ring may enroach on the orifice of the mitral valve leaflets and restrict their movements. While a supravalvular mitral ring may allow normal haemodynamic flow from the left atrium to the left ventricle, it often causes an obstruction of the mitral valve inflow [2]. It can occur as an isolated defect, but in nearly 90% of the patients, the supravalvular ring is found in conjunction with other congenital heart defects [3]. In the "Shone syndrome", it coexists with parachute mitral valvar, subvalvar aortic stenosis and aortic coartation. [4]

These two conditions have to be determined from the abnormal partition of the left atrium (cor triatriatum).

## Pathophysiology

The supravalvular mitral ring is a ridge or membrane arising from the left atrial wall overlying the mitral valve and is sometimes attached to the mitral valve anulus, variable in thickness and extension it can range from being a thin membrane to a thick fibrous ridge. The membranous variety may be difficult to detect, since the membrane often adheres to the anterior mitral valve leaflet while remaining just proximal to the posterior mitral leaflets [5]. In the same case, adhesion to the valve may impair opening movement of the leaflets, what's more, the ring may be large enough to protrude into the mitral valve inflow and cause obstruction. Sometimes, the ring may also be incomplete and eccentric, thereby, for an unobstructed flow through the mitral valve.

## Frequency

No data are available on the incidence of supravalvular mitral ring; in most patients it is detected during investigation for other congenital heart disease

No specific sex and race predilection exists [5,6].

## Case Report

**Figure 1:**
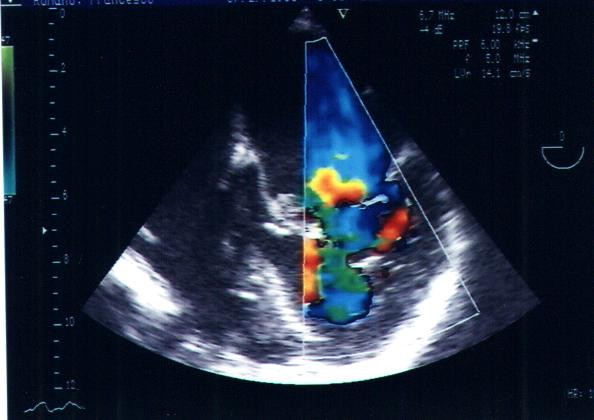
The transthoracic echocardiogram (TTE) showed left ventricular enlargment, atrial dilatation, moderate/severe mitral regurgitation and moderate aortic regurgitation mitral valve masses were suspected.

A 36-year-old male, was admitted to our Heart department: He experienced progressive dyspnea on effort and at rest. During the physical examination, he was found to have low blood pressure (90/60 mmHg), sinusal tachycardia and gallop rhythm. An olosistolic murmur was heard in the mitral area. Rales were available on pulmonary auscultation. The electrocardiogram (ECG) revealed sinus rhythm and left ventricular hypertrophy (Fig. 1).

Chest x-ray showed left atrial and ventricular enlargement; alveolar edema in the hilar regions of both lung fields.

The echocardiogram (TTE) showed left ventricular enlargment, atrial dilatation, moderate/severe mitral regurgitation and moderate aortic regurgitation; mitral valve masses were suspected.

Based on this diagnosis, the patient underwent a transesophageal-echo (TEE).

**Figure 2:**
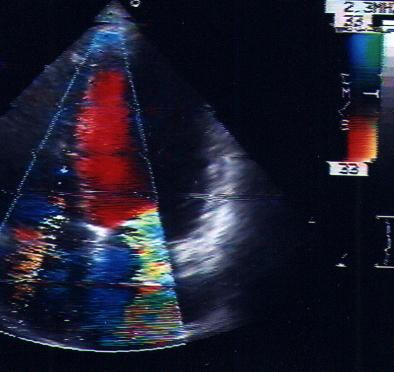
TEE showed, on apical 4-chamber section, an anular structure attached like a membrane to the atrial wall anterior mitral valve leaflet and just proximal to the posterior mitral leaflet.

**Figure 3:**
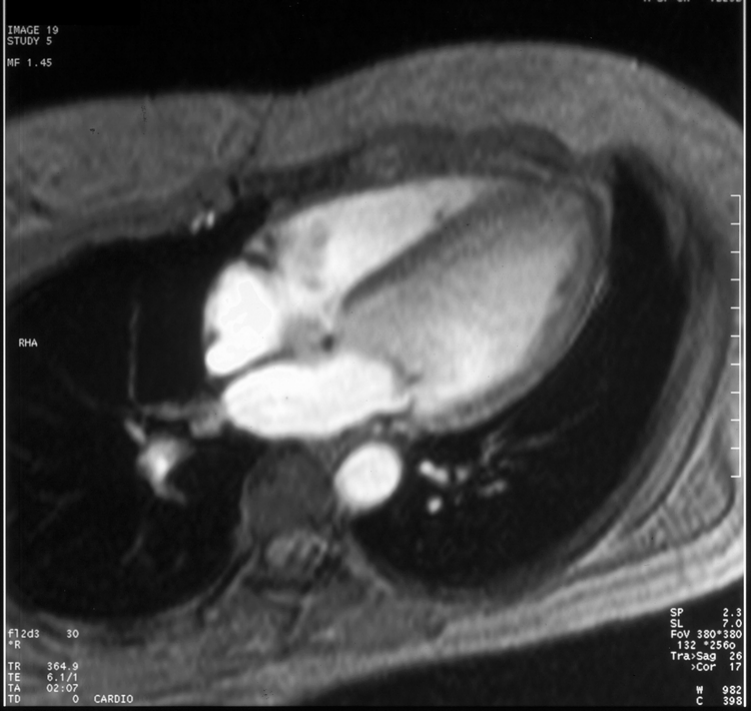
MRI cine turbo-flash images acquired on a 1.5 T scanner(Magnetom Vision-Siemens) in four-chamber (3A) and transverse plane, short-axis view (Fig. 4). These images didn't show congenital heart disease.

TEE showed, on apical 4-chamber section, an anulare structure attached from a membrane to the atrial wall anterior mitral valve leaflet and just proximal to the posterior mitral leaflet (Fig. 2, 3).

This supravalvular ring was proximal to the left atrial appendage, in contradistinction to "cor triatriatum"; it restricted the leaflets movement and impaired their opening. A severe mitral regurgitation (IV grade PISA) and mild diastolic gradient (6 mm/Hg DP mean) was detected by the Doppler-echocardiography (see additional file 1).

**Figure 4:**
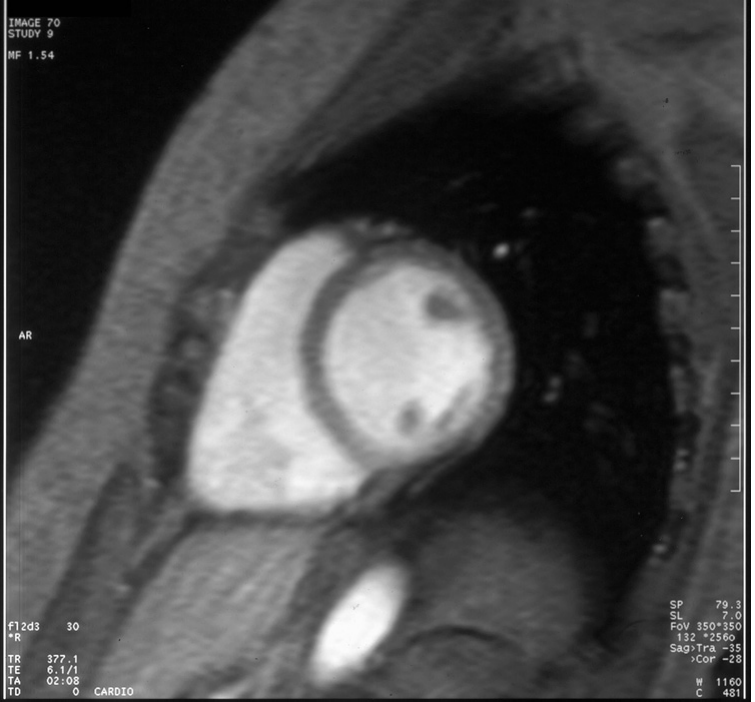
MRI cine turbo-flash images acquired on a 1.5 T scanner(Magnetom Vision-Siemens) in four-chamber (3A) and transverse plane, short-axis view. These images did n't show congenital heart disease.

A moderate/severe aortic regurgitation was seen. Mitral vegetations were not identified. Coronary angiography was normal; left cineventriculography showed a severe aortic and mitral regurgitation, but a mitral supravalvular structure was not noticed.

Based on TEE diagnosis, the patient underwent surgery in November 2002.

A left atriotomy enabled the identification of a membrane right above the mitral valve with 2 small openings that allowed blood to flow from the atrium to the left ventricle. The membrane was excised and the mitral and aortic valves were replaced. Follow up: after 6 month, the patient was in I NYHA class. The transtoracic echo showed a normal function of prostesis.

## Discussion

Supravalvular mitral ring rarely occurs as an isolated defect, and other congenital heart defects coexist in most (90%) patients [5,6]. The mitral valve itself is often abnormal and stenotic at the valvar or subvalvar level with fusion of leaflets, small valve orifice, and abnormal papillary muscle being common abnormalities. The Shone syndrome describes 4 congenital heart defects: mitral supravalvular ring, parachute mitral valve, subvalvar aortic stenosis and aortic coartation [7,8].

Obstruction to mitral inflow comes from reduction in the mitral valve orifice area. When significant, a diastolic pressure difference occurs between the left atrium and left ventricle and this haemodinamic condition causes, in severe cases, pulmonary edema.

Other common associated lesions in patients with supravalvular mitral ring are ventricular septal defect and tetralogy of Fallot [9].

Even in the rarest cases, such as the case described above, its occurrence can be isolated, as first described by Chung [10].

Pre-operative identification of the membrane is possible by TEE [11,12], where angiography often fails [13]. However, there are still many limitations in visualization of the membrane, that is usually very thin; in this case, we performed an MRI examination, but as described in other papers [14,15], there was a failure in the detection of the supravalvular mitral ring.

## Conclusion

Pre-operative identification of the supravalvular mitral ring is the target for obtaining good surgical results. Cineangiography and MRI both failed in reaching this objective, whereas, TEE is the best method to identify this congenital heart disease. Using TEE the identification is not only possible but also easier.

## Abbreviations

NYHA New York Heart Association

TTE Transthoracic echocardiography

TEE Transesophageal echocardiography

MRI Magnetic resonance imaging

## Authors' contributions

W. Serra has performed echocardiographic examinations for this article and has prepared the manuscript. P. Testa has performed the literature rewiew. All the authors have approved the final review of the manuscript.

We thank mr M. Conca, Techinician E-mail: maurizio.conca@unipr.it and mrs R. Bandini, librarian E-mail: rina.bandini@unipr.it

Department of Environmental Sciences University of Parma for having helped to prepare video-clips and to supply bibliographic research.

## Supplementary Material

Additional File 1 TEE showed, on apical 4-chamber section, an anulare structure attached like a membrane to the atrial wall anterior mitral valve leaflet and just proximal to the posterior mitral leaflet. This supravalvular ring was proximal to the left atrial appendage; a severe mitral regurgitation (IV grade PISA) and a mild diastolic gradient (6 mm/Hg DP mean) was detected by the Doppler-echocardiography. A moderate/severe aortic regurgitation was seen. Click here for file
